# Therapeutic Cancer Vaccines and Their Future Implications

**DOI:** 10.3390/vaccines11030660

**Published:** 2023-03-15

**Authors:** Randa Elsheikh, Abdelrahman M. Makram, Nguyen Tien Huy

**Affiliations:** 1 Deanery of Biomedical Sciences at Edinburgh Medical School, University of Edinburgh, Edinburgh EH8 9YL, UK; 2 School of Public Health, Imperial College London, London SW7 2BX, UK; 3 School of Tropical Medicine and Global Health, Nagasaki University, 1-12-4 Sakamoto, Nagasaki 852-8523, Japan

The continuous progress in vaccine development witnessed in the last decades, culminated with the development of vaccines against cancers, is set to change how various cancers are treated. Cancer vaccines can be divided into two main categories: prophylactic and therapeutic cancer vaccines.

Prophylactic vaccines are given to individuals at risk of developing certain types of tumors to reduce global disease morbidity and mortality [1]. Such vaccines have represented a breakthrough in preventing virus-induced tumors such as hepatocellular carcinoma [2] and cervical cancer [3]. Contrastingly, therapeutic cancer vaccines target existing malignancies and induce cancer regression by eliciting an immune anti-tumor response through tumor-associated antigens (TAAs) and tumor-specific antigens (TSAs) [4,5].

The first-ever therapeutic cancer vaccine dates back to 1980 when Hoover Jr et al. developed an autologous vaccine against colorectal cancer [6]. Subsequently, the development of a tumor antigen vaccine against melanoma represented the first use of tumor antigens in the treatment of cancers [7]. This was followed by Gardner et al.’s development of an autologous dendritic cells (DCs) vaccine for treating asymptomatic and minimally symptomatic prostatic cancer, which introduced the use of dendritic cells as a vaccine platform for anti-tumor vaccines development [8]. Recently, the advent of the COVID-19 pandemic and the race to produce mRNA vaccines opened the door for testing these vaccines in tumor therapy [9].

In addition to their solo use in cancer treatment, therapeutic cancer vaccines can be combined with other immunotherapies to achieve better results. For example, the combination of therapeutic vaccines with immune checkpoint blockade (ICB) has yielded promising results due to the prevention of T cell exhaustion by immune checkpoint molecules, thus potentiating the anti-tumor response [10]. An example is the combination of the GVAX tumor vaccine with ipilimumab, which has a more powerful immune response in pancreatic adenocarcinoma than when either therapy is used alone [11]. Furthermore, the discovery of neoantigens, generated from the mutation of tumor cells, has opened the door to personalized therapeutic cancer vaccines. These vaccines use next-generation sequencing to identify specific mutations in cancer patients, allowing them to tailor therapies to the mutated proteins. This elicits a more potent and lasting anti-tumor T-cell response, preventing tumor recurrence [12].

Four cell-based cancer vaccine platforms currently exist: peptide-based, nucleic acid-based, and virus-based (Figure 1).

Cell-based vaccines include tumor cell and immune cell vaccines. The former contains whole TAAs and is further classified into autologous or allogenic [1]. The expression of a large variety of TAAs makes the anti-tumor response non-specific, which prompts the use of adjuvants to potentiate vaccine immunogenicity. In cell-based vaccines, this can occur by combining them with radiotherapy, which enhances neutrophil recruitment and thus increases reactive oxygen species, eventually leading to tumor cell apoptosis and improving antigens recognition [13]. A well-known example of a cell-based vaccine is sipuleucel-T (Provenge), a DC-based vaccine used to treat advanced prostate cancer. This vaccine significantly improved the three-year survival of prostate cancer patients by increasing survival by a median of 4.5 months [14].

Virus-based vaccines include inactivated, live attenuated, and subunit vaccines and stimulate an anti-tumor response by triggering both innate and adaptive immune responses [4]. The most commonly used oncolytic viruses include herpes simplex virus and adenovirus [15]. The latter is futuristic due to its ability to be amended, its reproducibility, infectivity to the mucous membrane, and host cell tropism [16].

On the contrary, peptide-based vaccines are weaker in their immune response; therefore, adjuvants are mostly needed to enhance their immunogenicity [17]. These adjuvants can be antigen delivery systems [18] (that protect the antigenic particles from degradation, facilitate their uptake, and aid in their localization in lymph nodes) or immunopotentiators [19] (which act by enhancing innate immunity through the activation of pattern recognition receptors).

Lastly, nucleic acid vaccines are composed of a group of pathogen antigens (carriers) and the encoding gene. They can be in the form of ribonucleic acid (RNA), deoxyribonucleic acid (DNA) [20], or, most recently, mRNA, which can be used in combination with conventional cancer therapeutics to achieve a synergistic effect in improving clinical outcomes and/or defeating tumor resistance [4,21].

The success of mRNA vaccines in the fight against the COVID-19 pandemic has sparked hope in the potential anti-tumor effects of these vaccines. mRNA vaccines have proven to be an effective alternative to DNA, DC, and protein-based vaccines. This is because they have the advantage of being devoid of insertional mutagenesis, being able to encode for multiple antigens (and thus potentiating the anti-tumor response), and having better tolerability, fewer adverse effects, and the possibility of rapid, low-cost, and large-scale manufacturing. Moreover, they possess a higher protein expression rate than DNA vaccines, making them the nucleic acid vaccines with the highest future potential [22]. Nevertheless, owing to their ability to stimulate the interferon I system, mRNA vaccines could trigger an autoimmune reaction in predisposed individuals. This suggests special caution should be taken in screening at-risk individuals before vaccine administration [23].

Although therapeutic cancer vaccines represent an exciting frontier in the race to solve the age-old cancer treatment puzzle, identifying vaccine platforms that can achieve high immunogenicity is crucial. Moreover, addressing individual variations in tumor antigens is needed for better anti-tumor response.

In conclusion, future research should focus on improving immunogenicity by optimizing combination therapy and refining vaccine platforms for better clinical outcomes.

## Figures and Tables

**Figure 1:**
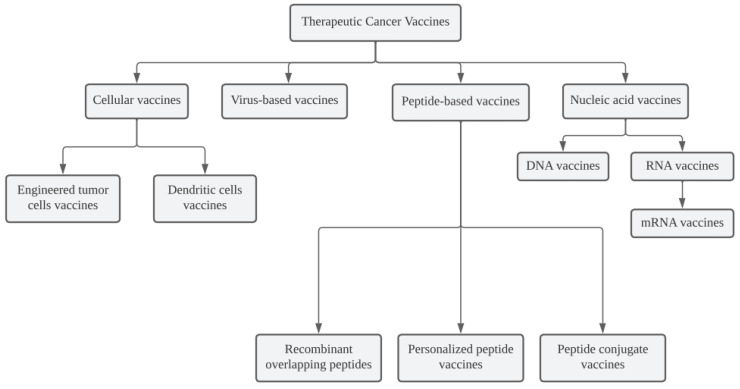
Used platforms for therapeutic cancer vaccines.
